# Assessment of Non-Phytate Phosphorus Requirements of Chinese Jing Tint 6 Layer Chicks from Hatch to Day 42

**DOI:** 10.3390/ani14142093

**Published:** 2024-07-17

**Authors:** Cheng-Yan Gong, Guang Liu, Hong-Peng Shi, Shuan Liu, Xin-Yi Gao, Shou-Jun Zhang, Hao Liu, Rui Li, Dan Wan

**Affiliations:** 1 Laboratory of Animal Nutritional Physiology and Metabolic Process, Key Laboratory of Agroecological Processes in Subtropical Region, Institute of Subtropical Agriculture, Chinese Academy of Sciences, Changsha 410125, China; gongchengyan21@mails.ucas.ac.cn (C.-Y.G.); liushuan18@mails.ucas.ac.cn (S.L.); xinyig0725@163.com (X.-Y.G.); 18395302256@163.com (S.-J.Z.); lh02282024@163.com (H.L.); 2 University of Chinese Academy of Sciences, Beijing 101408, China; shihongpeng1994@126.com; 3 Hubei Hongshan Laboratory, College of Animal Science and Technology, Huazhong Agricultural University, Wuhan 430070, China; lg19980905@163.com

**Keywords:** local chicken breed, available phosphorus requirement, growth performance, tibia, serum, phosphorus utilization

## Abstract

**Simple Summary:**

The non-phytate phosphorous (NPP) requirements of laying hens vary significantly at different stages of growth and development due to their differing physiological characteristics. Non-linear models and factorial methods are often used to estimate the nutritional requirements of animals at different stages of growth. In this study, the NPP requirement of Chinese Jing Tint 6 layer chicks from hatch to d 42 was evaluated using both a non-linear model and factorial method. The non-linear models were fitted based on the criterion of growth performance, serum biochemical parameters, bone characteristics, and phosphorus utilization. The factorial methods were used to classify the requirements of the layer chicks into growth requirements and maintenance requirements. Based on the current results, it was recommended that dietary 0.367% and 0.439% NPP benefit from the best P utilization of 1–14 and 15–42 d birds, respectively. By predicting the nutritional needs of poultry at different stages as accurately as possible, the model can help formulate more scientific and reasonable feed formulation.

**Abstract:**

We aimed to estimate the non-phytate phosphorus (NPP) requirements of Chinese Jing Tint 6 layer chicks. We randomly allocated 720 birds to five treatments with six cages of 24 birds each, feeding them a corn–soybean diet containing 0.36%, 0.41%, 0.46%, 0.51%, and 0.56% NNP. The results showed that the body weight gain (BWG), tibial length, and apparent total tract digestibility coefficients (ATTDC) of P were affected (*p* < 0.05) by dietary NPP level. A quadratic broken-line analysis (*p* < 0.05) of BWG indicated that the optimal NPP for birds aged 1–14 d was 0.411%. Similarly, 0.409% of NPP met tibial growth needs. However, 0.394% of NPP was optimal for P utilization according to the ATTDC criterion. For 15–42 d birds, 0.466% NPP, as estimated by the BWG criterion, was sufficient for optimal growth without decreasing P utilization. Using the factorial method, NPP requirements were calculated as 0.367% and 0.439%, based on the maintenance factors and BWG for 1–14 and 15–42 d birds, respectively, to maintain normal growth. Combining the non-linear model with the factorial method, this study recommends dietary NPP levels of 0.367% and 0.439% for 1–14 and 15–42 d birds, respectively, to optimize P utilization without affecting performance.

## 1. Introduction

Phosphorus (P) is an indispensable nutrient for birds and has important impacts on various physiological functions, especially in the formation of eggshells and skeletal development [1] and in working together with calcium to maintain bone mineralization [2]. Phosphorus deficiency may result in abnormal growth and development, bone diseases, digestive tract problems, impaired immune systems, and reduced eggshell quality in birds [3]. Most P in poultry diets is in the form of phytate P, and P availability in birds is less than 40% [4,5]. This necessitates dietary supplementation with inorganic P and exogenous phytase to meet the P requirements for optimal animal growth and production. However, several studies have shown increased P retention and utilization and reduced P excretion into the environment as a result of adding phytase to poultry diets [6,7]. However, the efficiency of exogenous phytases remains a challenge because it is affected by various physical, chemical, and biological factors [8]. Excess P is discharged in feces and retained in the soil, resulting in a decline in soil quality [9]. Therefore, to avoid excessive inorganic P in bird diets for maximal growth and bone development, the minimum P requirements of birds should be fully investigated.

Non-phytate P (NPP) recommendations for birds are mainly based on the National Research Council’s (NRC) guidelines [10]. The NPP requirements of laying hens vary significantly at different stages of growth and development due to their differing physiological characteristics [11,12,13]. In addition, most of the existing experiments were conducted with broilers, and it is known that the sex and breed of birds are two factors that influence their nutritional requirements [14,15]. Although layer chicks grow slower than broilers during the brooding period (1–42 d), this period is equally crucial for layer chicken farming. Adequate early nutrition is essential for the growth and health of layer chicks and their health and production performance in later stages [16,17,18].

Jing Tint 6 layer chicks are a breed based on Rhode Island Red and White Leghorn with high egg yield, low mortality, excellent egg quality, and modest body weight; as such, they make up more than 1/4 of the market for laying hens in China [19,20,21]. The amino acid requirements of Jing Tint 6 layer chicks aged 1–42 d of age have been well assessed [20]; however, micronutrient requirements, such as phosphorus, still need to be evaluated. Therefore, this study aimed to investigate the optimal dietary P levels in Jing Tint 6 layer chicks from hatching to d 42. Non-linear models based on the criteria of tibia length, growth performance, and serum parameters, combined with the factorial method, were used to obtain the most accurate estimates possible for the NPP requirements of Jing Ting 6 layer chicks.

## 2. Materials and Methods

This study was conducted following the guidelines of the National Act on the Use of Experimental Animals (People’s Republic of China). All animal procedures were approved by the Animal Welfare Committee of the Institute of Subtropical Agriculture, Chinese Academy of Sciences (IACUC # 201302).

### 2.1. Experimental Diets

Corn–soybean meal-based diets were formulated to meet the nutrient requirements according to the NRC (1994) [10] (Table 1). The NPP levels were 0.36, 0.41, 0.46, 0.51, and 0.56%, respectively. Samples of each diet were analyzed for calcium, crude protein, and total P following AOAC International (2016) [22]. Metabolizable energy (ME) and other dietary nutrient values were calculated according to the China Feed Database [23].

### 2.2. Birds and Housing

A total of 720 chicks procured from a commercial supplier (Beijing Huadu Yukou Poultry Industry Co., Ltd., Beijing, China) were weighed and randomly allocated to 5 treatments with 6 replicates of 24 birds per cage (90 cm deep × 600 cm wide × 40 cm high) at 1 d of age. Each cage was considered an experimental replicate, and each dietary treatment was fed to six replicates. The photoperiod was gradually reduced by 2 h per week from 24 to 12 h. The temperature was slowly decreased from 35 to 34 °C for 1–7 d, from 34 to 30 °C for 8–14 d, and from 30 to 24 °C for 15–42 d. The relative humidity was maintained at 65% with a 12 h/12 h light/dark cycle. During the experimental period, all birds had free access to feed and water, and the body weights and feed intake were recorded weekly.

### 2.3. Growth Performance

Experiments on NPP requirements were divided into two phases: phase 1, 1–14 d of age, and phase 2, 15–42 d of age. Feed consumption and the body weight of each cage were measured weekly, and the feed conversion ratio (FCR) was calculated.

### 2.4. Serum Biochemical Analysis

On d 14 and 42, 3 mL of blood samples were taken from a wing vein with a syringe from 2 birds close to the average weight randomly chosen from each cage, and serum was obtained via centrifugation at 3000 rpm at 4 °C for 10 min for biochemical analysis using an automated analyzer (Cobas c311, Basel, Switzerland) and commercial reagent kits (Lidman Biotech, Beijing, China). The serum biochemical indices included serum Ca, serum P, alkaline phosphatase (ALP), total protein (TP), and albumin (ALB).

### 2.5. Bone Characteristics

The same birds used for blood sampling were sacrificed, and their left tibias were collected and frozen at −20 °C until analyses were performed. The length and strength of the tibia were measured using an electronic universal testing machine (WDS-2000, Wenzhou, China).

### 2.6. Phosphorus Utilization

Titanium dioxide was incorporated into the diets (3 g/kg, as-fed) to calculate nutrient utilization using the index method. On d 10 and 38, excreta collection trays were introduced, and total excreta samples were collected during the last 3 d. Feathers and debris in the excreta were removed; it was sprayed with 10% hydrochloric acid and mixed well, and then stored at −20 °C. The collected excreta samples were thawed and mixed, dried to a constant weight at 56 °C, and ground to pass through a 0.5 mm screen before analysis. The collected excreta were analyzed for total P and Ti, which were then used to determine the apparent total tract digestibility coefficients (ATTDC) and endogenous P loss using Ti marker ratios in the diet and excreta [15,24].

### 2.7. Whole-Body Phosphorus Contents of Chickens (Carcass and Feathers)

On d 14 and 42, 2 chickens close to the average body weight were randomly selected from each cage. After weighing, the animals were euthanized using a non-bleeding asphyxiation method. The chickens were immediately scalded in hot water at 60–70 °C and then plucked. All feathers were collected in pre-weighed cotton bags, dried at 25 °C for 24 h, and then crushed to make feather samples for sealed storage. The gastrointestinal contents were discarded. The carcass was then ground using a meat grinder and mixed thoroughly before sufficient initial samples were collected and further homogenized at high speed. All samples (approximately 200 g) were collected and stored at −20 °C for further analysis. The P contents of the carcasses and feathers were analyzed to estimate the weight gain requirements using the factorial method.

### 2.8. Chemical Analysis

To determine the Ti concentration of the feed and fecal samples, inductively coupled plasma mass spectrometry (iCAPRQ, Schaumburg, IL, USA) was used according to the method described by AOAC International (2016) [22]. The P of the feed and fecal samples were determined using an inductively coupled plasma optical emission spectrometer (ICP-OES Optima 8000, Waltham, MA, USA), according to the method described by AOAC International (2016) [22]. The diets were analyzed for calcium and crude protein [22].

### 2.9. Calculations for Factorial Method

Calculation of P maintenance requirement:

P_M_ (mg/d) = (EP × DFI)/1000 × DM (1)

where P_M_ represents the P maintenance requirement, EP represents the endogenous P loss estimate on a DMI basis, DFI represents daily feed intake and DM represents dry matter percentage of feed.

Calculation of the P requirement for weight gain:

P_W_ (mg/d) = BWG × Pc (2)

where P_W_ represents P requirement for weight gain, BWG represents daily weight gain, and Pc represents carcass and feather P concentration.

Calculation of total net P requirement:

P_T_ (mg/d) = P_M_ + P_W_ (3)

where P_T_ represents the total net P requirement.

### 2.10. Statistical Analyses

All data were subjected to a one-way ANOVA using IBM SPSS Statistics 22 (Palo Alto, CA, USA), with the NPP level as the factor and the cage serving as the experimental unit for all statistical analyses. Regression analyses of non-linear models were performed in Origin 2023 (Northampton, MA, USA), and the best-fit models between the response criteria and dietary NPP levels were used to determine the dietary NPP requirements of the birds [25]. The level of statistical significance was set at *p* ≤ 0.05.

## 3. Results

### 3.1. Growth Performance

The growth performance of the birds during the 1–42 d period is provided in Table 2. Overall, at the end of 15 and 42 d of the study, there were no differences in daily feed intake (DFI) or FCR among the birds at different NPP levels (*p* > 0.05); however, there was a significant effect of diet on body weight gain (BWG) (*p* < 0.05), which showed a quadratic trend of first increasing and then decreasing with increasing dietary P levels (*p* < 0.05). For birds at 1–14 d, BWG was the highest in the birds fed 0.41% NPP, and for the birds at 15–42 d, BWG was the highest in the birds fed 0.46% NPP.

### 3.2. Tibia Characteristics

The effects of dietary treatments on tibial characteristics are provided in Table 3. A significant effect of diet was observed on tibial length (*p* < 0.05) for birds of 1–14 d, and tibial length was lowest in the birds fed 0.36% NPP. Tibial breaking strength increased with increasing P levels, but the difference was not significant (*p* > 0.05). At the age of 15–42 d, the 0.56% P treatment group had the longest tibia, which was significantly different from that of the other four groups (*p* < 0.05). A significant difference exists in the tibial breaking strength of the birds on d 42 between the 0.41% P treatment and 0.51% P treatment groups (*p* < 0.05).

### 3.3. Serum Parameters

As shown in Table 4, for serum parameters on d 14, there were no significant differences in the contents of TP, ALB, and ALP (*p* > 0.05). However, the diet had significant effects on serum calcium (Ca) and P (*p* < 0.05); the highest concentrations of Ca and P were both 0.41% NPP on d 14. The total protein concentration on d 42 showed a quadratic regression trend, first increasing and then decreasing with increasing P treatment levels (*p* < 0.05), with the highest point at 0.51% NPP. The ALB concentration on d 42 first increased and then decreased with increasing NPP levels (*p* < 0.05), with the highest point being observed in the 0.46% NPP group. The ALP and Ca contents on d 42 were not significantly different among the five groups. However, the P concentration on d 42 showed a decreasing linear regression trend with increasing P levels (*p* < 0.05).

### 3.4. Phosphorus Utilization

The dietary P intake, total P output, and apparent P digestibility of chicks fed different levels of NPP are shown in Table 5. The 0.41% P level had the highest apparent P digestibility at 14 d, which was significantly higher than that of the 0.56% NPP (*p* < 0.05). With increasing dietary NPP levels, apparent P digestibility showed a trend of first increasing and then decreasing. At 15–42 d of age, the 0.46% P treatment showed the highest apparent P digestibility (*p* < 0.05). The relationship between ingested P and excreted P, represented by dry matter intake (DMI), was calculated. At 14 d of age, the regression equation was y = 0.2158x + 1.3644 (R^2^ = 0.9704). Thus, the true utilization was 78.42%, and the endogenous P was 1.3644 g/kg DMI. The results at 15–42 d old showed that the regression equation was y = 0.4166x + 2.152 (R^2^ = 0.9548), which indicated that the true utilization was 58.34% and endogenous P was 2.152 g/kg DMI.

### 3.5. Estimated Available Phosphorus Requirements

#### 3.5.1. Non-Linear Model

The results of the available dietary P requirements of birds, as estimated by non-linear regression analyses, are shown in Table 6. The results indicated that BWG, tibial length, apparent P digestibility, and serum Ca and P were suitable criteria for evaluating the dietary NPP requirements of birds aged 1–14 d, and BWG, apparent P digestibility, and serum ALB and P were suitable for evaluating the dietary NPP requirements of birds aged 15–42 d. Based on the best-fitted straight broken line or quadratic broken line models of the above criteria, the optimal dietary NPP levels were estimated to be 0.449%, 0.409%, 0.394%, 0.452%, and 0.450% for birds fed a conventional corn–soybean meal diet from 1 to 14 days of age and 0.466%, 0.469%, 0.462%, and 0.422% for birds aged 15–42 days. However, the birds had the highest BWG at a dietary NPP level of 0.41% at 1–14 days of age and 0.46% at 15–42 d of age. Therefore, the NPP requirement would be approximately 0.41% to obtain the best growth rate and 0.45% to meet all P metabolism requirements of birds fed a conventional corn–soybean meal diet from 1 to 14 days of age. For the 15–42-day-old birds, 0.46% NPP was sufficient to meet all P metabolism requirements.

#### 3.5.2. Factorial Method

The NPP requirements for maintenance are listed in Table 7. The DFI was 10.56 g/d, and the DM percentage was 87.80%. Combined with the endogenous P of 1.36 g/kg DMI obtained in this study, the daily maintenance requirement of the experimental birds from 1 to 14 d of age was calculated as 12.61 mg/d. Using the same method, the daily maintenance requirement of the experimental birds aged 15–42 d was calculated as 52.06 mg/d.

The weight gain requirements of the birds at different stages are listed in Table 8. The P requirement for weight gain was (calculated by average P content in the carcasses and feathers × average weight gain) 26.13 mg/d and 68.88 mg/d for 1–14 d and 14–42 d of age, respectively.

A summary of the net maintenance and weight gain requirements is provided in Table 9. The total net physiological P requirements at 1–14 d and 15–42 d of age were 38.74 mg/d and 120.94 mg/d, respectively. According to the daily net P requirements and feed intake of birds at different stages, the NPP expressed as a percentage of diet for 1–14 d and 15–42 d was 0.367% and 0.439%, respectively.

## 4. Discussion

Phosphorus participates in many metabolic pathways [26,27,28] and plays an essential role in skeletal integrity [29,30]. In recent years, studies on phosphorus requirements have received considerable attention, and nutritionists have used various methods, including the dose–response method, factorial method, and non-linear mode, to obtain accurate requirements for different breeds and feeding stages [13]. However, the results regarding P requirements invariably differ across studies [11], which is partially attributed to the different breeds, diets, and methods used. In this study, non-linear models (tibia length, growth performance, and serum parameters) were combined with the factorial method to study the NPP requirements of birds and obtain the P requirements as accurately as possible.

A sufficient supply of P is critical for the growth and development of animals. In previous studies, growth performance was usually used as the target when studying the P requirements of birds [13,31]. However, the P availability differs when P levels vary among feeds. In low-P diets, birds improve their absorption and utilization efficiency of P while reducing the excretion of redundant P nutrients to adapt to the P levels [32]. In addition, the P levels available differ across diets, although the total P was consistent. Thus, the NPP levels in the diets were used in the current study, and the results showed that an increase in the dietary NPP level from 0.41% or 0.46% to 0.56% had a negative impact on the growth rate at the 1–14 d and 15–42 d stages. A possible reason for the negative effect of a high-NPP diet on pullet BWG may be that the relatively high NPP reduced Ca availability [33,34]. However, the phosphorus requirements in other studies vary. One study reported that birds achieved their greatest weight gain with 0.38% dietary NPP for 0–14 d broilers [35]. However, another study reported that a reduction in dietary NPP did not affect the growth performance of broilers at 0–28 d [36]. In the current study, the optimal dietary NPP was 0.41% for 1–14 d, and 0.46% for 15–42 d, indicating the most favorable growth performance.

P is an important component of bone and is essential for bone growth and density, as it can improve the mechanical strength of bones and reduce the occurrence of fractures. Studies have shown that providing an appropriate level of P improves the bone quality and sustainability of poultry production [1,13]. Tibial characteristics, such as breaking strength and length, have been traditionally used in birds to evaluate bone mineralization [13]. Compared with the concentrations of Ca or P, the ratio of Ca to NPP has been suggested to be more critical for the skeletal health of birds [37,38]. One study reported significant interactions between dietary Ca and NPP with the breaking strength and bone density of the tibia, which were improved when birds received 0.90% Ca and 0.40% NPP, or 1.00% Ca and 0.45% NPP [36]. In the current study, the NPP requirement of the 1–14 d birds was 0.41% based on tibia length.

Ca and P levels in serum are considered better parameters to reflect the nutritional status of NPP in birds [39,40]. The changes in Ca and P homeostasis are always indicated by the levels of serum Ca and P, which are essential for bone mineralization and normal physiology [37,41]. The current study observed that when the NPP level in the diet increased, serum P and Ca levels tended to increase in a quadratic manner. This indicates that an NPP level of 0.41% is sufficient to meet the pullet’s requirements, which is close to the results reported by Wang [36]. Ca or P deficiency interferes with both Ca and P homeostasis and impairs bone mineralization and growth rates [39,42]. Low serum P levels activate osteoclasts and decrease bone P levels to maintain P homeostasis. Additionally, serum ALB levels are a potential indicator of nutritional metabolism and health status. High dietary Ca and P levels result in a significant decrease in serum ALB [43]. Our results suggest that when the NPP level increased, the serum ALB level tended to increase in a quadratic manner, and the calculated NPP requirement for 15–42 d birds was 0.46% based on serum ALB.

Furthermore, the apparent P digestibility is used as a parameter to track the nutritional status of birds [44,45]. According to the studies by Liu et al. [15] and Xue et al. [46], increasing dietary P and Ca may decrease their apparent digestion rates. A similar decrease was observed in the current study. An increase in dietary NPP level from 0.41 or 0.46 to 0.56% had a negative impact on the apparent P digestibility of birds from 1–14 or 15–42 d. One study reported that the output of endogenous P was 0.73–0.81 g/kg DMI in broiler chickens aged 0–15 d by setting different Ca/P ratios [15]. These results are slightly lower than those of this study, and they may be related to the chicken breed, age in days, performance, P source, diet type, and other factors. Currently, few reports exist on the application of phosphate-free diets in China and abroad, and the exact measured value of the endogenous P output was not provided.

The methods used for nutritional requirement studies include the dose–response method (non-linear model), factorial method, and slaughter test method. Hurwitz et al. and Sun et al. have conducted numerous studies on the amino acid requirements of poultry using factorial methods [20,47]. The factorial analysis is a typical method for establishing dynamic models of nutrient requirements and can be used to dissect the components of nutrient requirements and nutrient utilization rates. As the tested parameters can be comprehensively applied, they are widely used to study nutritional requirements. In the current study, the individual body composition and growth performance of birds aged 1–42 d were analyzed using the factorial method, with results showing that the NPP requirements of 0–14 d and 15–42 d birds were 0.367% and 0.439%, respectively.

## 5. Conclusions

In conclusion, compared to the NRC (1994) [10] recommendations, which had an NPP requirement of 0.40% for 1–42 d birds and an NPP requirement of 0.46% for 1–10 d female broilers [11], the NPP requirement for layer chicks aged 8–21 d of age was 0.48% [48]. Here, fitted non-linear analysis using BWG, tibial length, ATTDC of P, serum Ca, and P content indicated that the optimal NPP for birds aged 1–14 d was 0.394–0.452%. Furthermore, among those aged 15–42 d, 0.422–0.466% NPP, as estimated by the corresponding criterion, was sufficient to meet all of the P metabolic needs without decreasing the utilization of P. In addition, using the factorial method, the NPP requirement to maintain normal growth was calculated as 0.367% and 0.439% for 1–14 and 15–42 d birds, respectively. By combining the non-linear model with the factorial method, the study recommended that dietary NPP of 0.367% and 0.439% results in the best growth performance of 1–14 and 15–42 d birds, respectively, without affecting P utilization. These values were higher than NRC recommendations and lower than those reported in previous studies.

## Figures and Tables

**Table 1:** Ingredient composition and nutrient values of the diets.

Ingredients, % “as-fed”	1–14 d (Levels of Non-Phytate P ^1^, %)					15–42 d (Levels of Non-Phytate P ^1^, %)				
	0.360	0.410	0.460	0.510	0.560	0.360	0.410	0.460	0.510	0.560
Corn	56.0	56.0	56.0	56.0	56.0	56.0	56.0	56.0	56.0	56.0
Soybean meal	35.1	35.1	35.1	35.1	35.1	35.1	35.1	35.1	35.1	35.1
Soybean oil	3.00	3.00	3.00	3.00	3.00	3.00	3.00	3.00	3.00	3.00
Wheat bran	0.800	0.800	0.800	0.800	0.800	0.800	0.800	0.800	0.800	0.800
Limestone	2.08	1.81	1.53	1.25	0.970	2.08	1.81	1.53	1.25	0.970
Mono-calcium phosphate	2.00	2.27	2.55	2.83	3.11	2.00	2.27	2.55	2.83	3.11
Salt	0.300	0.300	0.300	0.300	0.300	0.300	0.300	0.300	0.300	0.300
DL-Methionine	0.140	0.140	0.140	0.140	0.140	0.140	0.140	0.140	0.140	0.140
L-lysine HCl	0.140	0.140	0.140	0.140	0.140	0.140	0.140	0.140	0.140	0.140
V Premix1 ^2^	0.040	0.040	0.040	0.040	0.040	0.040	0.040	0.040	0.040	0.040
Mineral Premix ^3^	0.300	0.300	0.300	0.300	0.300	0.300	0.300	0.300	0.300	0.300
Total amount	100	100	100	100	100	100	100	100	100	100
Calculated nutritional level										
ME ^4^ (Mcal/kg)	2.90	2.90	2.90	2.90	2.90	2.92	2.92	2.92	2.92	2.92
Crude protein (%)	20.0	20.0	20.0	20.0	20.0	19.0	19.0	19.0	19.0	19.0
Total P (%)	0.740	0.780	0.840	0.890	0.940	0.740	0.780	0.840	0.890	0.940
Lysine (%)	2.62	2.62	2.62	2.62	2.62	2.62	2.62	2.62	2.62	2.62
Methionine (%)	0.590	0.590	0.590	0.590	0.590	0.590	0.590	0.590	0.590	0.590
Tryptophan (%)	0.0200	0.0200	0.0200	0.0200	0.0200	0.0200	0.0200	0.0200	0.0200	0.0200
Threonine (%)	1.64	1.64	1.64	1.64	1.64	1.64	1.64	1.64	1.64	1.64
Analyzed nutritional level										
Crude protein (%)	19.6	19.6	19.6	19.6	19.6	18.3	18.3	18.3	18.3	18.3
Calcium	1.05	1.05	1.05	1.05	1.05	1.08	1.08	1.08	1.08	1.08
Total P (%)	0.92	1.02	1.09	1.14	1.2	0.92	1.02	1.09	1.14	1.2

^1^ The non-phytate P was calculated by subtracting dietary phytate P from dietary total P. The NPP values were designed with reference to the NRC (1994). ^2^ Vitamin premix provided per kg of diet: vitamin A, 11,000 IU; vitamin D3, 3000 IU; vitamin E, 20 IU; vitamin K, 3 mg; vitamin B12, 0.02 mg; riboflavin, 6.5 mg; folic acid, 1 mg; calcium pentothenate, 10 mg; niacin, 39.9 mg; biotin, 0.2 mg; thiamine, 2.2 mg; pyridoxine, 4.5 mg; choline, 1000 mg. ^3^ Mineral premix provided per kg of diet: Mn (manganese dioxide), 66 mg; Fe (ferrous sulfate), 80 mg; Cu (copper sulfate), 10 mg; Se (sodium selenite), 0.3 mg; I (calcium iodate), 0.4 mg; and sodium chloride (salt), 0.67 mg. ^4^ ME = metabolizable energy.

**Table 2:** Effect of dietary NPP level on growth performance of birds aged 1–42 d.

Phase	Variables ^1^	Non-Phytate P, %					Pooled SEM ^2^	*p*-Value ^3^		
		0.360	0.410	0.460	0.510	0.560		Available P	L	Q
14 d	Final BW, g/bird	119 ^ab^	122 ^a^	113 ^b^	113 ^b^	111 ^b^	1.52	0.056	0.014	0.746
	BWG, g/bird/day	5.71 ^ab^	5.94 ^a^	5.15 ^b^	5.10 ^b^	5.07 ^b^	0.10	0.003	<0.001	0.007
	DFI, g/bird/day	10.8	10.6	11.4	10.7	11.4	0.27	0.839	0.513	0.897
	FCR, g/g	1.86	1.83	2.09	1.85	2.10	0.06	0.331	0.205	0.881
42 d	Final BW, g/bird	359 ^b^	404 ^a^	408 ^a^	385 ^ab^	368 ^ab^	6.70	0.042	0.025	0.005
	BWG, g/bird/day	8.58 ^b^	10.1 ^ab^	10.4 ^a^	9.60 ^ab^	9.19 ^ab^	0.22	0.015	0.195	0.005
	DFI, g/bird/day	22.1	27.6	25.8	24.3	26.6	1.12	0.602	0.488	0.524
	FCR, g/g	2.47	2.47	2.42	2.74	2.92	0.14	0.842	0.905	0.825

^1^ Final BW = final body weight; DFI, daily feed intake; BWG, body weight gain; FCR, feed conversion ratio. We recorded the number of birds per cage simultaneously with feed intake and body weight. ^2^ SEM = standard error of the mean. ^3^ Non-phytate P = treatment effect; L = linear; Q = quadratic. ^a,b^ Values within a row with different superscripts differ significantly at *p* < 0.05.

**Table 3:** Effect of dietary available phosphorus on tibial characteristics of birds on d 14 and 42.

Variables	Phase	Non-Phytate P, %					Pooled SEM ^1^	*p*-Value ^2^		
		0.360	0.410	0.460	0.510	0.560		Available P	L	Q
Length, mm	14 D 42 d	43.5 ^b^	45.3 ^a^	45.2 ^a^	44.9 ^a^	44.7 ^a^	0.15	0.002	0.027	<0.001
		55.3 ^b^	57.0 ^b^	56.1 ^b^	51.6 ^b^	64.3 ^a^	0.08	<0.001	0.030	0.010
Strength, kg	14 d 42 d	2.35	2.44	2.52	2.25	2.17	1.04	0.720	0.740	0.840
		7.35 ^ab^	9.86 ^a^	8.10 ^ab^	6.01 ^b^	7.40 ^ab^	0.36	0.010	0.090	0.320

^1^ SEM = standard error of the mean. ^2^ Non-phytate P = treatment effect; L = linear; Q = quadratic. ^a,b^ Values within a row with different superscripts differ significantly at *p* < 0.05.

**Table 4:** Effect of dietary available phosphorus on serum biochemistry of birds on d 14 and 42.

Phase	Variables ^1^	Non-Phytate P, %					Pooled SEM ^2^	*p*-Value ^3^		
		0.36	0.41	0.46	0.51	0.56		Available P	L	Q
14 d	TP, g/L	34.4	35.1	30.8	29.8	30.7	0.80	0.120	0.030	0.560
	ALB, g/L	18.8	18.2	16.7	15.7	17.1	0.46	0.210	0.070	0.240
	ALP, U/L	8049	7434	10,148	8621	6610	771	0.830	0.980	0.380
	Ca, mmol/L	3.13 ^ab^	3.43 ^a^	2.70 ^ab^	2.63 ^b^	2.80 ^ab^	0.09	0.009	0.005	0.039
	P, mmol/L	2.98 ^ab^	3.21 ^a^	2.54 ^b^	2.60 ^b^	2.35 ^b^	0.09	0.010	0.000	0.710
42 d	TP, g/L	34.0	35.5	35.4	36.7	31.8	0.64	0.140	0.460	0.030
	ALB, g/L	16.8 ^b^	17.8 ^b^	20.1 ^a^	17.6 ^b^	15.4 ^b^	0.41	0.000	0.190	0.000
	ALP, U/L	1127	1228	1194	1908	1375	149	0.490	0.280	0.680
	Ca, mmol/L	2.92	2.86	2.85	3.01	2.85	0.02	0.150	0.920	0.820
	P, mmol/L	2.72 ^a^	2.59 ^ab^	2.54 ^ab^	2.43 ^b^	2.33 ^b^	0.04	0.010	0.000	0.990

^1^ TP = total protein; ALB = albumin; ALP = alkaline phosphatase. ^2^ SEM = standard error of the mean. ^3^ Non-phytate P = treatment effect; L = linear; Q = quadratic. ^a,b^ Values within a row with different superscripts differ significantly at *p* < 0.05.

**Table 5:** Dietary phosphorus intake, total phosphorus output, and P apparent total tract digestibility coefficients from birds fed graded levels of available phosphorus.

Phase	Variables ^1^	Non-Phytate P, %					Pooled SEM ^2^	*p*-Value ^3^		
		0.360	0.410	0.460	0.510	0.560		Available P	L	Q
14 d	P intake, g/kg DMI ^1^	10.2	11.1	11.8	12.5	13.1	-	-	-	-
	P output, g/ kg DMI ^1^	3.59	3.73	3.93	5.71	6.48	0.37	0.00	0.00	0.03
	ATTDC, %	62.8 ^ab^	68.0 ^a^	59.2 ^abc^	54.3 ^b^	50.7 ^c^	1.91	0.00	0.00	0.11
42 d	P intake, g/ kg DMI ^1^	10.1	11.5	12.0	12.0	11.9	-	-	-	-
	P output, g/ kg DMI ^1^	6.34	7.02	7.16	9.06	8.59	0.46	0.36	0.1	0.78
	ATTDC, %	34.5	38.7 ^ab^	49.2 ^a^	33.2 ^ab^	27.8 ^b^	2.42	0.03	0.06	0.01

^1^ DMI = dry matter intake; ATTDC = apparent total tract digestibility coefficients. ^2^ SEM = standard error of the mean. ^3^ Non-phytate P = treatment effect; L = linear; Q = quadratic. ^a–c^ Means within a row lacking a common superscript differ significantly (*p* < 0.05).

**Table 6:** Estimations of dietary NPP requirements of birds aged 1–42 d using a prediction model.

Phase	Dependent Variable ^1^	Regression Equation	R^2^	*p*-Value	Requirements ^2^
1–14 d	Body weight gain	y = −26.0771 + 161.7267X − 203.9962X^2^	0.577	0.001	0.411
	Tibial length	y = 32.8185 + 29.8404X	0.492	0.002	0.409
	ATTDC	y = −559.5571 + 3130.0748X − 3900.9427X^2^	0.762	0.008	0.394
	Serum Ca	y = −30.3980 + 169.7561X − 212.8219X^2^	0.471	0.004	0.452
	Serum P	y = −25.6906 + 145.3867X − 182.6667X^2^	0.323	0.004	0.450
15–42 d	Body weight gain	y = 1.7731 + 8.2290X − 29.6305X^2^	0.257	0.045	0.466
	ATTDC	y = −210.3100 + 1137.7635X − 1275.5178X^2^	0.507	0.024	0.469
	Serum albumin	y = 7.2332 + 26.6820X	0.376	0.007	0.462
	Serum P	y = 4.7845 − 5.45001X	0.286	0.013	0.422

^1^ ATTDC = apparent total tract digestibility coefficients. ^2^ Predicted requirements = requirements of dietary non-phytate P, %.

**Table 7:** Estimations of phosphorus requirements for maintenance of birds aged 1–42 d.

Phase	Endogenous P Loss, g/kg of DMI	Feed Intake, g/d	DM, %	Maintenance Requirements, mg/d
1–14 d	1.36	10.6	87.8	12.6
15–42 d	2.15	27.6	87.8	52.1

**Table 8:** Estimations of phosphorus requirements for weight gain of birds aged 1–42 d.

Phase	Average Weight, g	Average Weight Gain, g/d	Average P Content ^1^, %	Requirements, mg/d
1–14 d	122	5.94	0.440	26.1
15–42 d	404	10.1	0.680	68.9

^1^ Average P content = average P content in the carcasses and feathers.

**Table 9:** Estimations of NPP requirements of birds aged 1–42 d using the factorial method.

Parameter	1–14 d	15–42 d
P maintenance requirements (mg/d)	12.6	52.1
P weight gain requirements (mg/d)	26.1	68.9
Net P requirement (mg/d)	38.7	120.9
Feed intake (g/d)	10.6	27.6
Factorial requirement of NPP (%)	0.367	0.439

## Data Availability

Data are contained within the article.
